# Editorial: Feeding and nutritional strategies to reduce livestock greenhouse gas emissions: Volume II

**DOI:** 10.3389/fvets.2022.1101468

**Published:** 2023-01-05

**Authors:** Manuel Gonzalez-Ronquillo, Paula Toro-Mujica

**Affiliations:** ^1^Departamento de Nutricion Animal, Facultad de Medicina Veterinaria y Zootecnia, Universidad Autonoma del Estado de Mexico, Instituto Literario 100 Ote, Toluca, Mexico; ^2^Instituto de Ciencias Agroalimentarias, Animales y Ambientales, Universidad de O'Higgins, San Fernando, Chile

**Keywords:** enteric methane mitigation, ruminant, secondary metabolites, chemical methanogenic inhibitors, electron acceptors, essential oils

In this editorial, we present a compilation of articles related to feeding and nutrition strategies to reduce greenhouse gas emissions from livestock.

Livestock and manure management are significant contributors to agricultural greenhouse gas (GHG) emissions, accounting for ~18% of global GHG production (1). In order to reduce this contribution of GHG from ruminates, various mitigation strategies have been proposed. These mitigation strategies related to greenhouse gases in ruminants have been classified according to their mitigation approach in two parts. On the one hand, we have the reduction of total emissions (inhibition of methane production in the rumen), and on the other hand, the reduction of CH_4_ per unit of production, with the reduction of emissions intensity.

Among the practices that reduce the total emissions is the use of antimethanogenic substances, which can be classified as methanogenic chemical inhibitors. These can be divided into “specific” inhibitors, which include structural analogs of coenzyme M and HMG-CoA inhibitors, and “non-specific” inhibitors, which include chemical substances that inhibit the activity of methanogens and non-methanogens (2) such as ionophores (i.e., monensin, lasalocid), electron acceptors (NO-3NO_3_-), and plants containing secondary metabolites (tannins, saponins, essential oils, and terpenoids). These products have been used as additives for ruminants, showing an effect proportions of volatile fatty acids and methane synthesis in the rumen (3). Dietary essential oils (i.e., garlic, thyme, clove, orange peel, mint, cinnamon, etc.) have been used widely, as well as other plants containing secondary metabolites (i.e., *Cymbopogon citratus, Matricaria chamomilla*, and *Cosmos bipinnatus)* (4).

Practices such as selecting high-quality feed, intensive housing, use of rotational grazing to sequester carbon in the soil, increasing diet digestibility (5), selecting the type of carbohydrates in the diet, increasing reproductive efficiency, and breeding for higher productivity, have been proposed to reduce emission intensity and improve animal health and welfare (Figure 1).

**Figure 1:**
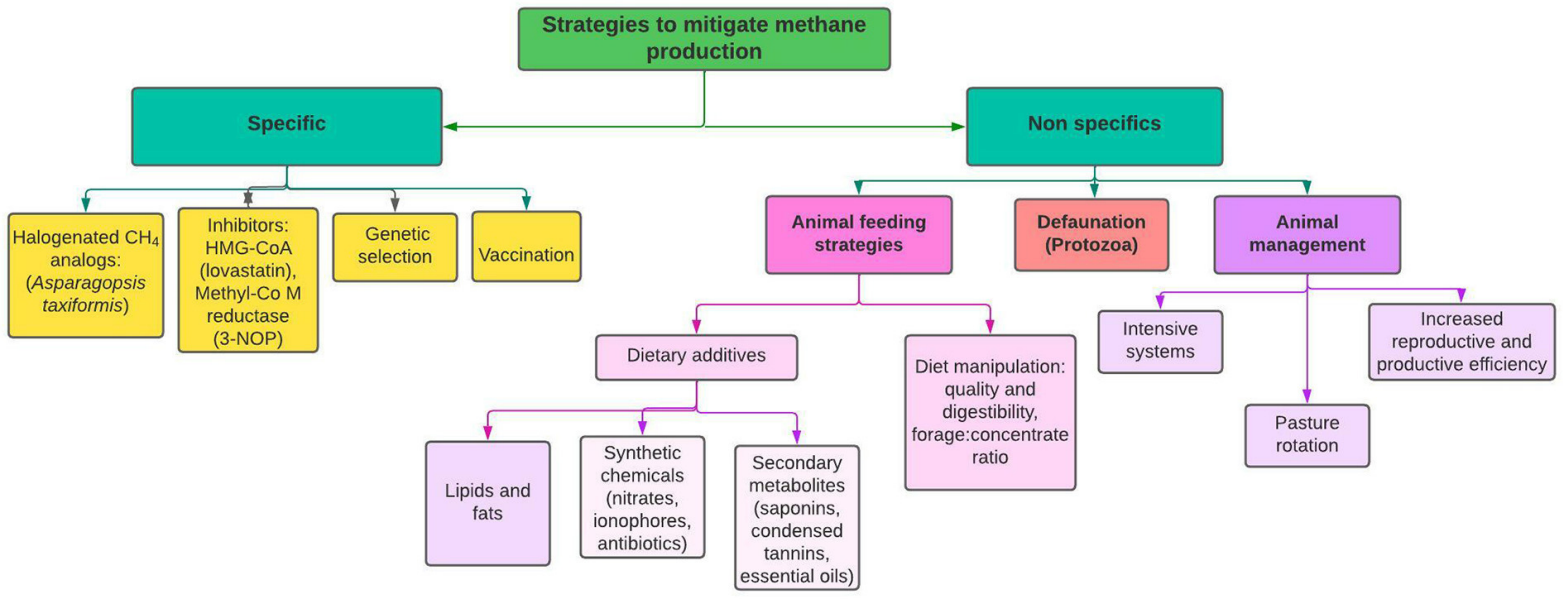
Potential strategies for reducing enteric methane production in ruminants.

There are four articles showing some methane mitigation strategies in the present Research Topic.

The type of carbohydrate fermentation and the carbohydrates derived at the rumen level must be determined in the evaluation of the mitigation potential. Sun et al. review the types of carbohydrates (monosaccharides, disaccharides, oligosaccharides, and polysaccharides), showing that these affect the concentration of dissolved hydrogen at the rumen level. This alters the fermentation pathways, resulting in differences in CH_4_ emissions. Rhamnose is the only monosaccharide that produces low CH_4_ emissions; on the other hand, Pectic polysaccharides present a higher CH_4_ production with respect to other carbohydrates. This is due to the conversion of methyl groups (due to their degree of esterification) into methanol and finally into CH_4_.

Methane production by methanogens at the ruminal level represents a digestive inefficiency for the ruminants, this can represent a loss of 12% of the gross energy consumed, in this sense, strategies that promote the flow of reducing substrates produced during fermentation out of methanogenesis and toward energetically favorable alternative electron sinks have been sought (6). One alternative is the administration of supplemental nitrate (NO-3NO_3_-) to diets. In this regard, Božic et al. use intraruminal administration of 120 mg nitroethane/kg LW/day in cows to transiently reduce the methane-producing activity in rumen fluid up to 3.6-fold, while, on the other hand, the methane-producing activity in feces increased (8.8 times) compared to pretreatment measurements.

In another study, Jiménez-Ocampo et al. examine the effects of orange essential oil (OEO) on ruminal fermentation with a bermudagrass (Cynodon dactylon) diet and find that supplementation of 0.5% OEO reduces CH_4_ emissions by 12% (g/day) without negatively impacting feed intake.

Recently, the inclusion of algae in the diet has been considered a strategy to reduce methane emissions (7). From this perspective, Choi et al. study five different species of red algae namely *A. anceps, A. taxiformis, C. tenellus, G. elliptica*, and *G. parvispora*. At the *in vitro* level, the results showed that Succinivibrionaceae, Anaeroplasma, and Ruminococcaceae, are associated with higher propionate production. Furthermore, at the *in vitro* level, the results showed that Anaeroplasma, Succinivibrionaceae, and Ruminococcaceae are associated with increased propionate production, due to higher amylolytic activity and, consequently, to a higher starch degradation in the red algae extracts with respect to the control group. This indicates that supplementation with red algae extracts alters the microbiota, increasing propionate production and reducing CH_4_ production, propionate being the final fermentation product of several bacterial species, including organisms of the Propionbacteriaceae family. Propionic acid is the only volatile fatty acid with gluconeogenic activity and the potential to improve the efficiency of metabolizable energy utilization in the whole animal (8).

Therefore, the alternative strategies include the induction of changes in the ruminal microbiome, which can be through the inclusion of plants containing secondary metabolites (4), the direct addition of essential oils, or the inclusion of red algae species. These oils and metabolites induce alterations in ruminal fermentation, re-channeling H_2_ toward more energy-efficient biochemical pathways (i.e., propionate synthesis) that will decrease CH_4_ formation and make energy use more efficient for the ruminant in its different physiological stages.

Finally, all the strategies outlined above must support sustainability (for people, planet, and profitability) and ethics in order to be more environmentally friendly, and we must examine the consequences of current and future strategies for animal welfare and contrast them with their effectiveness in mitigating climate change.

## Author contributions

Both authors listed have made a substantial, direct, and intellectual contribution to the work and approved it for publication.
